# Black phosphorus-based van der Waals heterostructures for mid-infrared light-emission applications

**DOI:** 10.1038/s41377-020-00356-x

**Published:** 2020-07-02

**Authors:** Xinrong Zong, Huamin Hu, Gang Ouyang, Jingwei Wang, Run Shi, Le Zhang, Qingsheng Zeng, Chao Zhu, Shouheng Chen, Chun Cheng, Bing Wang, Han Zhang, Zheng Liu, Wei Huang, Taihong Wang, Lin Wang, Xiaolong Chen

**Affiliations:** 1 Department of Electrical and Electronic Engineering, Southern University of Science and Technology, 518055 Shenzhen, China; 2 Key Laboratory of Flexible Electronics (KLOFE) & Institute of Advanced Materials (IAM), Jiangsu National Synergetic Innovation Center for Advanced Materials (SICAM), Nanjing Tech University (Nanjing Tech), 30 South Puzhu Road, 211816 Nanjing, China; 3 Key Laboratory of Low-Dimensional Quantum Structures and Quantum Control of Ministry of Education, and Key Laboratory for Matter Microstructure and Function of Hunan Province, Hunan Normal University, 410081 Changsha, China; 4 Department of Materials Science and Engineering, Southern University of Science and Technology, 518055 Shenzhen, China; 5 Center for Programmable Materials School of Materials Science and Engineering Nanyang Technological University, Singapore, 639798 Singapore; 6 Institute of Microscale Optoelectronics, Collaborative Innovation Centre for Optoelectronic Science & Technology, Key Laboratory of Optoelectronic Devices and Systems of Ministry of Education and Guangdong Province, College of Physics and Optoelectronic Engineering, Shenzhen Key Laboratory of Micro-Nano Photonic Information Technology, Guangdong Laboratory of Artificial Intelligence and Digital Economy (SZ), Shenzhen University, 518060 Shenzhen, China; 7 Frontiers Science Center for Flexible Electronics (FSCFE), Shaanxi Institute of Flexible Electronics (SIFE) & Shaanxi Institute of Biomedical Materials and Engineering (SIBME), Northwestern Polytechnical University (NPU), 127 West Youyi Road, 710072 Xi’an, China

**Keywords:** Inorganic LEDs, Optical properties and devices

## Abstract

Mid-infrared (MIR) light-emitting devices play a key role in optical communications, thermal imaging, and material analysis applications. Two-dimensional (2D) materials offer a promising direction for next-generation MIR devices owing to their exotic optical properties, as well as the ultimate thickness limit. More importantly, van der Waals heterostructures—combining the best of various 2D materials at an artificial atomic level—provide many new possibilities for constructing MIR light-emitting devices of large tuneability and high integration. Here, we introduce a simple but novel van der Waals heterostructure for MIR light-emission applications built from thin-film BP and transition metal dichalcogenides (TMDCs), in which BP acts as an MIR light-emission layer. For BP–WSe_2_ heterostructures, an enhancement of ~200% in the photoluminescence intensities in the MIR region is observed, demonstrating highly efficient energy transfer in this heterostructure with type-I band alignment. For BP–MoS_2_ heterostructures, a room temperature MIR light-emitting diode (LED) is enabled through the formation of a vertical PN heterojunction at the interface. Our work reveals that the BP–TMDC heterostructure with efficient light emission in the MIR range, either optically or electrically activated, provides a promising platform for infrared light property studies and applications.

## Thin-film semiconductors enhance black phosphorus mid-infrared light emission

Layering thin films of semiconducting transition metal dichalcogenides with thin-film black phosphorus enhances its ability to emit mid-infrared (MIR) light, which could facilitate MIR light emission investigations and applications. Xiaolong Chen of China’s Southern University of Science and Technology and colleagues layered thin flakes of black phosphorus (BP) with one or the other of a monolayer of tungsten diselenide (WSe_2_) or a thin film of molybdenum disulfide (MoS_2_). Thin-film BP is considered a promising MIR material, with potential applications in MIR photodetection and optical modulation. WSe_2_ efficiently transferred light energy to the BP, enhancing its MIR photoluminescence by up to 192%. On the other hand, the interface created by layering BP with MoS_2_ enabled the formation of a MIR light-emitting diode at room temperature.

## Introduction

As an emerging member of the two-dimensional (2D)-layered material family, black phosphorus (BP)^1–6^ has been widely studied for its unique properties, such as in-plane anisotropy^5,7^, infrared bandgap energy^8–10^, and high carrier mobility^11–14^, which enable wide applications in electronic and optoelectronic devices^15,16^. Due to the efficiently tuneable bandgap energy through thickness (0.3–2 eV) and electric-field (down to 0.05 eV)^17–21^ modulation, thin-film BP is considered a promising mid-infrared (MIR) material, filling the energy gap between semimetallic graphene and semiconducting transition metal dichalcogenides (TMDCs) (1.0–2.5 eV)^22^. Utilizing the MIR properties of thin-film BP, optoelectronic devices, such as MIR photodetectors and optical modulators with high performance, have been demonstrated^17,22–27^.

A decent light-emission property is also crucial for photonic and optoelectronic device applications. Previous reports have focused on the visible and near-infrared photoluminescence (PL) properties of monolayer and few-layer BP (<5 layers)^9,28–31^. Until very recently, the MIR PL of thin-film BP was investigated by Chen et al., revealing that thin-film BP is a promising material for MIR light-emission applications^8^. To achieve higher efficiency and lower power-consumption devices, thin-film BP with a better light-emission density is necessary. In addition, more emission driving modes are also desirable. For example, electrically driven MIR light emission (electroluminescence/EL) is more favorable for practical photonic and optoelectronic applications.

Due to the high-quality interface and lack of lattice mismatch, vdWs heterostructures built from 2D-layered materials, such as graphene and TMDCs, have been intensively investigated for various applications, including transistors^32^, solar cells^33^, photodetectors^34^, and light-emitting devices^35^. In recent years, BP-based vdWs heterostructures have begun to attract great attention due to their narrow bandgap and anisotropic lattice structure. For example, BP–MoS_2_ heterostructures have enabled high-performance photodetectors^36^ and high-gain logic inverters^37–39^. BP–graphene heterostructures can sustain a large pseudomagnetic field at the interface^40^.

Here, we introduce a high-quality van der Waals (vdWs) heterostructure targeted for MIR light-emission applications constructed from thin-film BP and TMDCs, such as monolayer tungsten diselenide (WSe_2_) and thin-film molybdenum disulfide (MoS_2_). Combining density functional theory (DFT) calculations^41^ and experimental observations, a type-I band alignment is formed in the BP–WSe_2_ heterostructure, and efficient energy transfer from WSe_2_ to thin-film BP is enabled. As a result, a 192% enhancement of the MIR PL is observed at a wavelength of 2.79 μm, and this enhancement effect persists up to 3.89 μm. On the other hand, a PN heterojunction tuneable by a source–drain voltage is achieved in the BP–MoS_2_ heterostructure, with which an MIR light-emitting diode (LED) is demonstrated. In addition, highly anisotropic PL and EL are observed in these BP–WSe_2_ and BP–MoS_2_ heterostructures, respectively. All the results suggest that constructing BP–TMDC heterostructures is an efficient and facile strategy for MIR light-emission investigations and applications.

## Results

### Mid-infrared photoluminescence enhancement in BP–WSe_2_ heterostructures

**Fig. 1:**
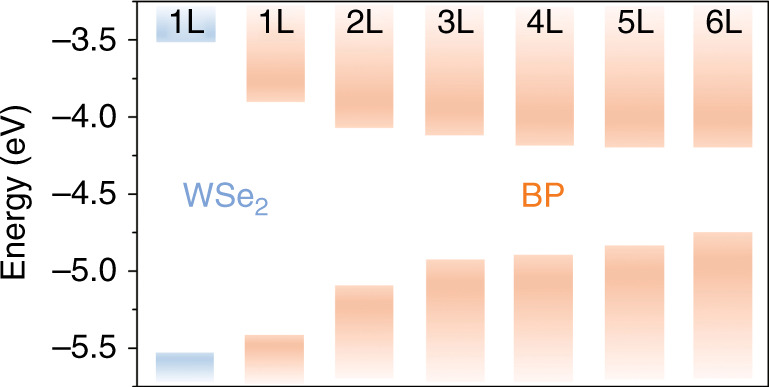
Density functional theory investigation of BP–WSe_2_ heterostructures. Band diagram of monolayer WSe_2_ and BP (with layer numbers from one to six) calculated by the HSE06 functional. Type-I band alignment is formed in the BP–WSe_2_ heterostructures

Figure 1 shows the DFT-calculated electron affinity and bandgap size of monolayer WSe_2_ and BP. The detailed band structures are further illustrated in Supplementary Fig. 1. The bandgap size of BP decreases with increasing thickness, consistent with previous theoretical calculations and experimental observations^6,15,41^. In addition, BP shows a direct bandgap at all thicknesses, suggesting that it is a promising material for light-emission applications. To realize its application in the MIR region (2.5–25 μm), we select thin-film BP with a layer number larger than six to construct the heterostructure. According to the DFT calculation, the BP–WSe_2_ heterostructure forms a type-I band alignment. In this band alignment, thin-film BP serves as a quantum well and can efficiently collect electron and hole pairs from adjacent monolayer WSe_2_^42^. On the other hand, monolayer WSe_2_ has a high optical absorption and excellent quantum efficiency in the visible region (complementary to the MIR range of thin-film BP)^43^. Hence, it is an ideal optical absorption layer for enhancing the luminescence efficiency of BP in terms of the absorption wavelength, recombination efficiency, and energy transfer.

**Fig. 2:**
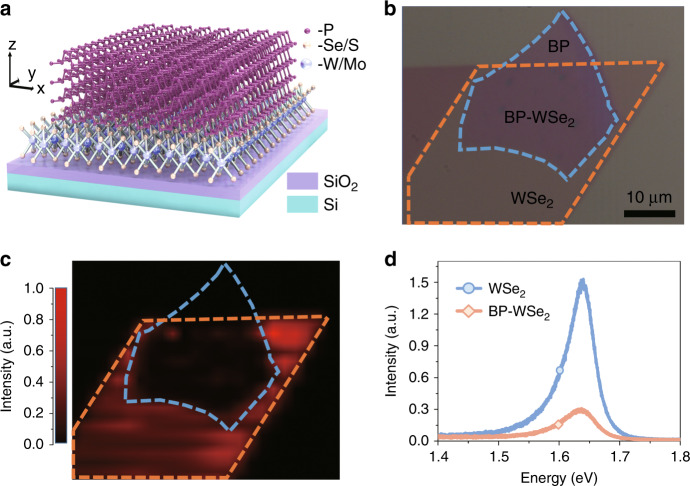
Configuration and visible photoluminescence of BP–WSe_2_ heterostructures. **a**–**c** Schematic (**a**), optical (**b**), and photoluminescence (PL) mapping (**c**) images of the BP–WSe_2_ heterostructure. The scale bar is 10 μm. **d** PL spectra of monolayer WSe_2_ and the BP–WSe_2_ heterostructure under an incident laser of 2.33 eV. The incident laser power is 4 μW

Figure 2a shows a schematic diagram of the BP–TMDC heterostructure. The bottom TMDC layer has a hexagonal crystalline structure, with a layer of tungsten/molybdenum atoms sandwiched between two selenium/sulfur atom layers^22^. The phosphorus atoms in each layer of BP form a folded anisotropic honeycomb structure^15^. The *x*- and *y* directions are used to denote the armchair and zigzag crystalline directions of BP, respectively. To build this heterostructure, first, TMDC flakes and thin-film BP were mechanically exfoliated onto 285-nm SiO_2_/Si and polydimethylsiloxane (PDMS) substrates, respectively. Then, the thin-film BP was transferred onto the TMDCs using the PDMS-assisted transfer method^44^. To enhance the vdWs interactions between BP and the TMDCs, the heterostructures were further heated at a temperature of 200 °C for 10 min. All operations were performed in a glovebox filled with nitrogen to avoid BP surface oxidation and achieve a high-quality vdWs interface. In this work, the BP flakes were aligned with the TMDC layers with random angles, and all the samples showed PL enhancement in the MIR range. However, the alignment angle between BP and the TMDCs can possibly influence the detailed emission properties of the heterostructure, which is outside the scope of this article. Further studies on this effect are highly encouraged and will benefit the heterostructure 2D materials community. Figure 2b shows an optical image of a typical BP–WSe_2_ heterostructure sample. The contours of the monolayer WSe_2_ and thin-film BP flakes are outlined by orange and blue dashed lines, respectively. The thickness of thin-film BP is ~6 nm, as determined by atomic force microscopy (AFM) and PL measurements (see Supplementary Fig. 2). The Raman spectra of the BP–WSe_2_ stack show the characteristic vibration modes of both thin-film BP and monolayer WSe_2_ (see Supplementary Fig. 3), indicating successful preparation of the heterostructure.

We first examined the PL properties of the BP–WSe_2_ heterostructure in the visible region under 2.33- eV laser excitation. Figure 2c shows the PL emission mapping of the sample at room temperature. High PL intensity is observed in the monolayer WSe_2_ region due to its high light absorption and quantum efficiency, consistent with previous studies^45,46^. On the other hand, significant PL quenching is observed in the BP–WSe_2_ heterojunction area. As shown in Fig. 2d, the PL intensity in the heterostructure region decreases by 80% compared with that of monolayer WSe_2_. This observation agrees with our theoretical prediction of the type-I band alignment between monolayer WSe_2_ and BP, which enables efficient carrier transfer from the wide bandgap WSe_2_ to the narrow bandgap BP. As a result, the photogenerated electron and hole densities in WSe_2_ decrease, leading to a significant PL intensity reduction in the heterostructure region. Another argument is that the PL intensity reduction for WSe_2_ in the heterostructure region is due to the light absorption of the top BP layer. To exclude this possibility, we assembled another BP–WSe_2_ heterostructure under ambient conditions with a 7.5-nm-thick BP flake. Due to the reaction of BP with water and oxygen during the fabrication process under ambient conditions, a thin phosphorus oxide (P_x_O_y_ ~ 2 nm) will form at the BP–WSe_2_ interface, resulting in a BP–P_x_O_y_–WSe_2_ three-layer structure^20,47^. The interface oxide layer can effectively prevent charge transfer between BP and WSe_2_, and hence, a small reduction of the visible PL intensity should be observed in the heterostructure region. In this device, only a 33% reduction of the PL intensity is observed in the heterostructure region (see Supplementary Fig. 4), indicating that the contribution from the light absorption by the top BP layer is insignificant.

**Fig. 3:**
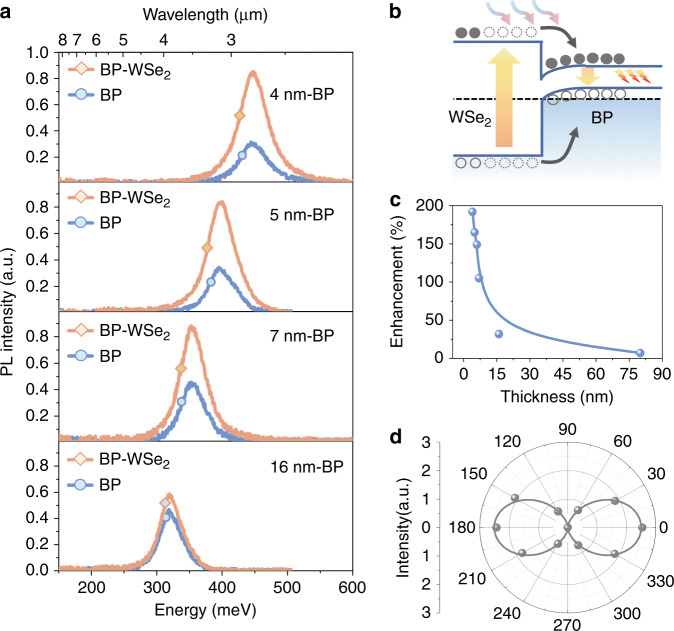
MIR photoluminescence spectra of BP–WSe_2_ heterostructures. **a** MIR PL spectra of thin-film BP (blue lines) and BP–WSe_2_ heterostructures (orange lines) at 80 K. **b** Schematic band diagram of BP–WSe_2_ heterostructures. The dashed line denotes the Fermi energy of BP and WSe_2_. **c** MIR PL enhancement in BP–WSe_2_ heterostructures as a function of thickness. The solid line is a guide line. **d** Polarization-resolved MIR PL spectra for the BP–WSe_2_ heterostructure with 5-nm-thick BP. The solid line is the fitting curve obtained using the equation *I*= (*I*_max_ − *I*_min_) cos^2^*θ* + *I*_min_. Here, *θ* is the polarization angle referenced to the armchair axis of BP, and *I*_max_ and *I*_min_ are the PL intensities along the armchair- and zigzag axes, respectively

To further demonstrate the efficient energy transfer in the BP–WSe_2_ heterostructure, we characterized its MIR light-emission properties under 2.33-eV laser excitation. The incident laser power was fixed at 20 μWμm^2^ with a laser spot diameter of ~15 μm. PL studies at additional incident laser powers are shown in Supplementary Fig. 5. Figure 3a shows the MIR PL spectra of the BP film and BP–WSe_2_ heterostructure region, where the BP thickness is approximately 5 nm. We achieve a 165% enhancement of the MIR PL intensity in the heterostructure region, which is defined as (*I*_BP-WSe2_ − *I*_BP_)/*I*_BP_. Here, *I*_BP-WSe2_ and *I*_BP_ are the MIR PL intensities of the BP and BP–WSe_2_ heterostructure regions, respectively. In addition, the PL peaks of the two regions are at the same position of ~3.18 μm, indicating that carriers transferred from monolayer WSe_2_ are effectively confined in the BP quantum well and then recombined with MIR light emission, as illustrated in Fig. 3b. For the BP–P_x_O_y_–WSe_2_ heterostructure sample fabricated in air, the enhancement at the heterostructure is only ~25%. This is due to the presence of the phosphorus oxide at the interface, which significantly reduces the carrier transfer rate from monolayer WSe_2_ to BP. In addition, we characterized the enhancement of the MIR PL intensity at additional laser-excitation energies. Enhancement factors of 138% and 116% are achieved at excitation energies of 1.95 eV and 1.81 eV, respectively, as shown in Supplementary Fig. 6. We attribute the excitation energy-dependent enhancement factor to the optical absorption of monolayer WSe_2_. A higher absorption can generate more electron–hole pairs and thus result in a larger enhancement factor. At a laser-excitation energy of 1.58 eV, which is below the exciton energy of monolayer WSe_2_, no enhancement effect is observed in the heterostructure region. This phenomenon provides direct evidence that type-I band alignment is achieved in the BP–WSe_2_ heterostructure.

We further performed MIR PL measurements of BP–WSe_2_ heterostructures with BP thicknesses ranging from 4 nm to 80 nm at 80 K. As shown in Fig. 3a, c, we achieve a PL enhancement over a broad MIR region from 2.79 μm to 3.89 μm. For example, a 192% enhancement is obtained in the heterostructure with 4-nm-thick BP (see Fig. 3c). For the 16-nm-thick BP, the enhancement is ~32%. The PL enhancement of the heterostructures gradually decreases as the thickness of BP increases. This phenomenon can be attributed to two reasons. On the one hand, the MIR emission from the BP–WSe_2_ heterostructure is contributed by two components (*I* = *I*_s_ + *I*_t_). The component *I*_s_ comes from the self-generated electron–hole pairs in BP, and *I*_t_ comes from the electron–hole pairs transferred from monolayer WSe_2_. It is obvious that higher *I*_t_ and smaller *I*_s_ will lead to a larger enhancement effect. For thicker BP flakes, the ratio between *I*_t_ and *I*_s_ decreases because thicker BP absorbs more light and the bottom WSe_2_ layer absorbs less light. On the other hand, the band bending of BP near the BP–WSe_2_ interface can separate the electrons and holes, as shown in Fig. 3b. Thicker BP flakes will lead to a larger spatial separation between electrons and holes and will hence decrease the electron–hole recombination efficiency. As a result, the enhancement factor is reduced for thicker BP flakes. We also plot the MIR PL peak position of the BP–WSe_2_ heterostructures as a function of BP thickness and temperature, and the results are in good agreement with previous reports on thin-film BP^8^ (see Supplementary Figs. 7 and 8).

The highly anisotropic MIR light emission is still preserved in the BP–WSe_2_ heterostructures, as demonstrated by the polarization-resolved PL spectra shown in Fig. 3d. Here, the laser-excitation direction is fixed along the armchair axis of BP, and the detection angle *θ* is the intersection angle between the detection direction and the armchair axis of BP.

### Mid-infrared electroluminescence in BP–MoS_2_ heterojunction diodes

In contrast to the BP–WSe_2_ heterostructure, the BP–MoS_2_ heterostructure forms a type-II band alignment according to previous experimental observations^36,37^. In addition, thin-film BP and MoS_2_ always show p-type and n-type semiconducting characteristics, respectively, due to the presence of defects^1,2,39,48^. As a result, a PN heterojunction is naturally formed at the BP–MoS_2_ interface, and a diode can be built from this heterostructure. Through tuning of the source–drain voltage, the transfer of electrons from the MoS_2_ conduction band to the BP conduction band is possible, enabling electrically driven MIR light emission in BP.

**Fig. 4:**
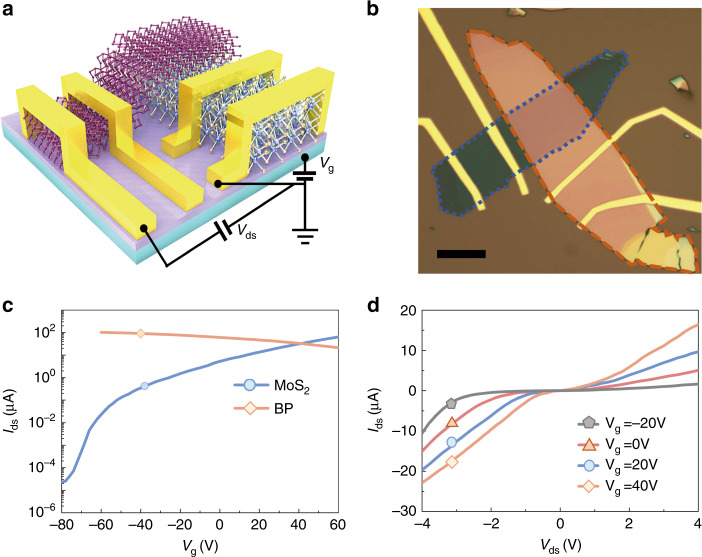
Configuration and electrical characterization of the BP–MoS_2_ heterojunction diode. **a**, **b** Schematic (**a**) and optical (**b**) images of the BP–MoS_2_ heterojunction diode. The scale bar is 10 μm. MoS_2_ and BP flakes are enclosed by blue and orange dashed lines, respectively. **c** Transfer curves of thin-film MoS_2_ (blue line) and BP (orange line) at source–drain voltage *V*_ds_ = 0.5 V at room temperature. **d** Source–drain current *I*_ds_ as a function of *V*_ds_ at various gate voltages *V*_g_ for the BP–MoS_2_ heterojunction diode at room temperature

Figure 4a, b shows a schematic and optical images of a BP–MoS_2_ heterojunction diode, respectively. The thickness of the BP flake is ~60 nm, as determined by AFM (see Supplementary Fig. 2). We chose 7-nm-thick MoS_2_ instead of a monolayer, taking advantage of the higher carrier mobility in thin-film MoS_2_^32^. We first characterized the transport properties of thin-film BP and MoS_2_. As demonstrated by the transfer curves in Fig. 4c, thin-film BP and MoS_2_ exhibit p-type and n-type characteristics, respectively. The weak gate tuneability of the BP conductance can be attributed to the larger thickness of ~60 nm, which will screen the gate-field effect. The linear drain–source current–voltage curves (*I*_ds_ − *V*_ds_) indicate that ohmic contacts are achieved between the metal electrodes (Cr/Au 5/60 nm) and 2D flakes (see Supplementary Fig. 9).

**Fig. 5:**
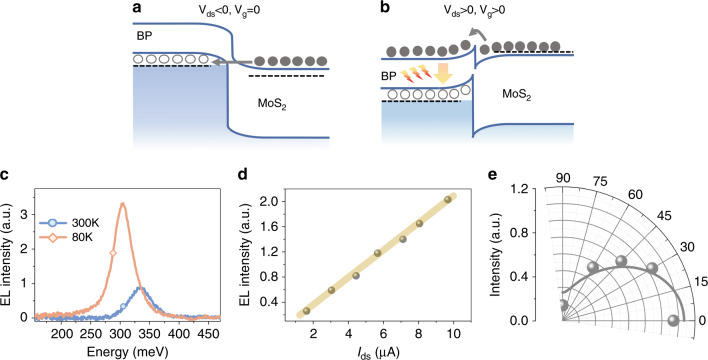
Mid-infrared electroluminescence in the BP–MoS_2_ heterojunction diode. **a**, **b** Schematic band diagram of the BP–MoS_2_ heterojunction diode for *V*_ds_ < 0, *V*_g_ = 0 (**a**) and *V*_ds_ > 0, *V*_g_ > 0 (**b**). **c** EL at 80 K, *I*_ds_ = 8.05 μA (orange line) and at 300 K, *I*_ds_ = 8.50 μA (blue line). **d** EL intensity as a function of source–drain current *I*_ds_ when *V*_ds_ > 0. The yellow solid line serves as a guide line. **e** Polarization-resolved EL emission at 80 K and *I*_ds_ = 8.05 μA. The solid line is the fitting curve obtained using the equation *I* = (*I*_max_ − *I*_min_) cos^2^*θ* + *I*_min_. Here, *θ* is the polarization angle referenced to the armchair axis of BP, and *I*_max_ and *I*_min_ are the EL intensities along the armchair- and zigzag-directions, respectively

The *I*_ds_−*V*_ds_ characterization of the BP–MoS_2_ heterojunction diode at room temperature is shown in Fig. 4d. Under a negative *V*_ds_, band-to-band tunneling of charge carriers from the MoS_2_ conduction band to the BP valence band is possible (see Fig. 5a). Hence, a large current is observed under negative *V*_ds_. This band-to-band tunneling phenomenon has also been observed in previous studies of BP-ReS_2_, BP-SnSe_2_, and BP–MoS_2_ heterostructures^39,47,49^. Under a positive *V*_ds_, the conduction band of MoS_2_ bends downward, and electrons accumulate near the MoS_2_ surface, while the valence band of BP bends upward, and holes accumulate near the BP surface (see Fig. 5b). Since the barrier height is lowered for *V*_ds_ > 0, thermionic electrons from the conduction band of MoS_2_ to that of BP are enabled. On the other hand, holes in BP are confined near the BP surface due to the larger barrier height (~1 eV). As a result, active electron and hole recombination will occur in thin-film BP. In addition, the drain–source current *I*_ds_ can be further tuned by the gate voltage *V*_g_. For a positive *V*_g_, the electron concentrations in MoS_2_ are higher, leading to a larger *I*_ds_, while the Fermi energy of BP is less affected by *V*_g_ due to the electron screening in MoS_2_.

The EL spectra of the BP–MoS_2_ heterojunction diode at 80 K and 300 K are shown in Fig. 5c. At 80 K, the EL spectrum is maximized at a wavelength of 4.09 μm. Importantly, the EL still persists at room temperature. The abnormal blueshift of the EL spectra at higher temperatures can be attributed to the temperature-induced strain effect in BP, consistent with previous observations of temperature-dependent PL spectra of thin-film BP^8^. The intensity of the EL spectra shows a good linear relation with the injected current, as shown in Fig. 5d. Similar to the PL spectra of BP, the EL spectra also show highly anisotropic characteristics. The EL intensity reaches the maximum (minimum) value when the detection direction is along the armchair axis (zigzag-axis) of BP. The EL intensity ratio between the armchair axis and zigzag-axis is over 7 (see Fig. 5e).

## Discussion

In summary, we have shown that BP–TMDC heterostructures (BP–WSe_2_ and BP–MoS_2_) are promising candidates for MIR light-emission applications. For the BP–WSe_2_ heterostructures, a type-I band alignment is formed, and a large enhancement of the PL intensities in the MIR region is observed due to the efficient energy transfer from WSe_2_ to BP. In addition, the PL of the BP–WSe_2_ heterostructures also shows strong polarization and thickness dependences. For the BP–MoS_2_ heterostructures, the type-II band alignment enables the formation of a PN heterojunction at the interface. Based on this heterostructure, an MIR LED has been further realized at room temperature, which fills a gap in the research field of 2D-material-based LEDs.

## Materials and methods

### Theoretical method

Density functional theory (DFT) calculations were performed using the generalized gradient approximation (GGA) of Perdew–Burke–Ernzerhof (PBE) as implemented in the Vienna Ab initio Simulation Package (VASP). The hybrid Heyd−Scuseria−Ernzerhof (HSE06) functional was selected to calculate the band structure and band alignments of BP–TMDC heterostructures. The optB88-vdW functional correction was used to describe the long-range vdWs interaction. A cutoff energy of 400 eV was set for the plane wave expansion. The convergence criterion of energy was set to 10^−6^ eV, and that of the force on each atom was less than 0.01 eV Å^−1^. The vacuum layer height along the *z* direction was set to 15 Å to avoid interactions between two adjacent images. Monkhorst–Pack k-point grids of 12 × 12 × 1 and 10 × 12 × 1 were used in the first Brillouin zone for WSe_2_ and BP, respectively.

### Sample preparation

TMDC flakes were prepared on a 285-nm-SiO_2_/Si substrate with the standard mechanical exfoliation method in an atmospheric environment. Meanwhile, thin-film BP was mechanically exfoliated from bulk BP crystals onto a PDMS/glass substrate. BP was then overlaid onto the WSe_2_ flake by the PDMS-assisted dry transfer technique under an optical microscope in a glovebox^44^. The heterostructure samples were further heated at a temperature of 200 °C for 10 min in the glovebox to increase the vdWs interactions between BP and the TMDC flakes. To prevent oxidation and photodegradation^50^, all processes involving BP were performed in a nitrogen-filled glovebox.

### Optical characterizations

For visible PL measurements, the samples were placed in a vacuum chamber. PL and Raman spectroscopies were performed in a confocal HORIBA LabRAM system equipped with 600 grooves per millimeter gratings. Related measurements were carried out at room temperature using a ×50 objective, and the incident laser was 532 nm with a power fixed at 4 μW. Due to the glass layer between the objective lens and the sample, the diameter of the laser spot focused on the sample was 2–4 μm. For MIR PL and EL spectroscopies, samples were placed on a low-temperature stage coupled with a Bruker FTIR spectrometer and a Hyperion 2000 microscope. The MIR PL and EL signals were collected using the lock-in scheme as reported in previous studies^9^, which can significantly reduce the random thermal noise from the environment. For MIR PL measurement, a 533-nm incident laser was chopped at a frequency of 10 kHz, and the laser spot size on the sample was ~15 μm. The incident power was fixed at 20 μWμm^−2^. A Stanford Research SR830 was used to lock the frequency and coupled to the FTIR spectrometer. For MIR EL measurement, a sinusoidal voltage with a frequency of 1 kHz and a peak-to-peak voltage of 30 V was applied to the gate of the BP–MoS_2_ heterojunction diode. As a result, the EL spectra were modulated to an AC signal, and random thermal noise could be filtered by the lock-in amplifier. To reduce the effect of CO_2_ absorptions at an MIR wavelength of 4.3 μm, the system was purged with N_2_ gas for 1 h before PL and EL measurements (see Supplementary Fig. 10).

## Supplementary information

Supplementary Information

## Data Availability

The data that support the findings of this study are available from the corresponding author upon reasonable request.
