# Supplementary material for: Black phosphorus-based van der Waals heterostructures for mid-infrared light-emission applications
Source: Light Sci Appl. 2020 Jul 2;9:114. doi: 10.1038/s41377-020-00356-x (PMC7329856; doi:10.1038/s41377-020-00356-x)
Supplement: Supplementary file 1 — Supplementary Information [file 41377_2020_356_MOESM1_ESM.docx]

**Supplementary Information for**

**Black phosphorus-based van der Waals heterostructures for mid-infrared light emission applications**

Xinrong Zong^1,2,†^, Huamin Hu^3,†^, Gang Ouyang^3,†^, Jingwei Wang^4^, Run Shi^4^, Le Zhang^1^, Qingsheng Zeng^5^, Chao Zhu^5^, Shouheng Chen^1^, Chun Cheng^4^, Bing Wang^6^, Han Zhang^6^, Zheng Liu^5^, Wei Huang^2,7^, Taihong Wang^1,*^, Lin Wang^2,*^, Xiaolong Chen^1,*^

*^1^Department of Electrical and Electronic Engineering, Southern University of Science and Technology, Shenzhen 518055, China*

*^2^Key Laboratory of Flexible Electronics (KLOFE) & Institute of Advanced Materials (IAM), Jiangsu National Synergetic Innovation Center for Advanced Materials (SICAM), Nanjing Tech University (Nanjing Tech), 30 South Puzhu Road, Nanjing 211816, China.*

*^3^Key Laboratory of Low-Dimensional Quantum Structures and Quantum Control of Ministry of Education, and Key Laboratory for Matter Microstructure and Function of Hunan Province, Hunan Normal University, Changsha 410081, China*

*^4^Department of Materials Science and Engineering, Southern University of Science and Technology, Shenzhen 518055, China*

*^5^Center for Programmable Materials School of Materials Science and Engineering Nanyang Technological University, Singapore 639798, Singapore*

*^6^Institute of Microscale Optoelectronics, Collaborative Innovation Centre for Optoelectronic Science & Technology, Key Laboratory of Optoelectronic Devices and Systems of Ministry of Education and Guangdong Province, College of Physics and Optoelectronic Engineering, Shenzhen Key Laboratory of Micro-Nano Photonic Information Technology, Guangdong Laboratory of Artificial Intelligence and Digital Economy (SZ), Shenzhen University, Shenzhen 518060, China*

*^7^Frontiers Science Center for Flexible Electronics (FSCFE), Shaanxi Institute of Flexible Electronics (SIFE) & Shaanxi Institute of Biomedical Materials and Engineering (SIBME), Northwestern Polytechnical University (NPU), 127 West Youyi Road, Xi'an 710072, China*

^†^*These authors contributed equally to this article*

^*^Correspondence authors:

Xiaolong Chen

Email: [chenxl@sustech.edu.cn](mailto:chenxl@sustech.edu.cn)

Telephone: +86-755-88018260

Fax: +86-755-88018261

Lin Wang

Email: [iamlwang@njtech.edu.cn](mailto:iamlwang@njtech.edu.cn)

Telephone: +86-025-83587982

Taihong Wang

Email: wangth@sustech.edu.cn

Telephone: +86-755-88018258

Fax: +86-755-88018259


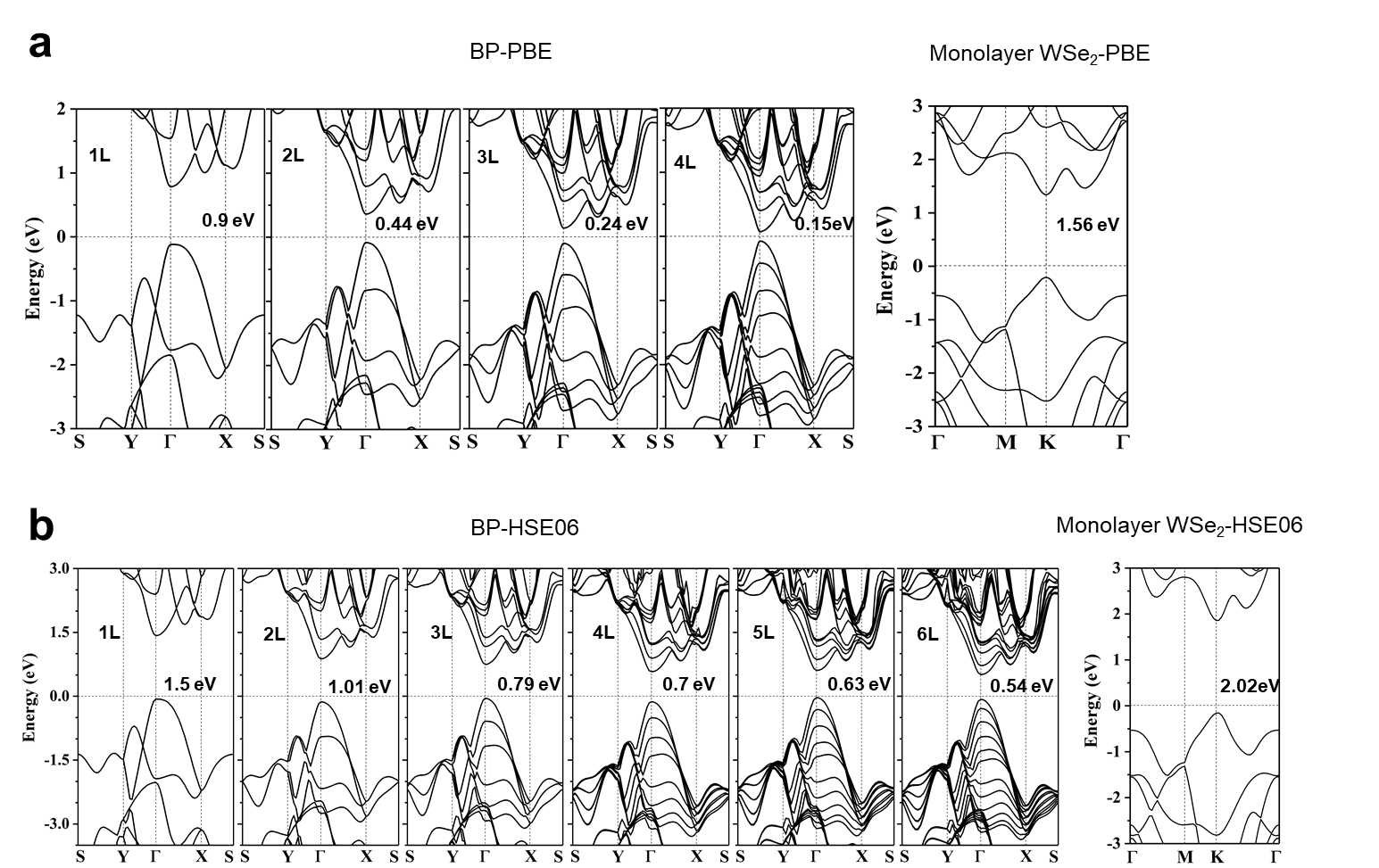


**Figure S1 | DFT calculations of electronic structure of TMDC and BP**. **a**, **b**, Band structures of monolayer WSe_2_ and thin-film BP calculated with PBE (a) and HSE06 (b) functions^1-3^.


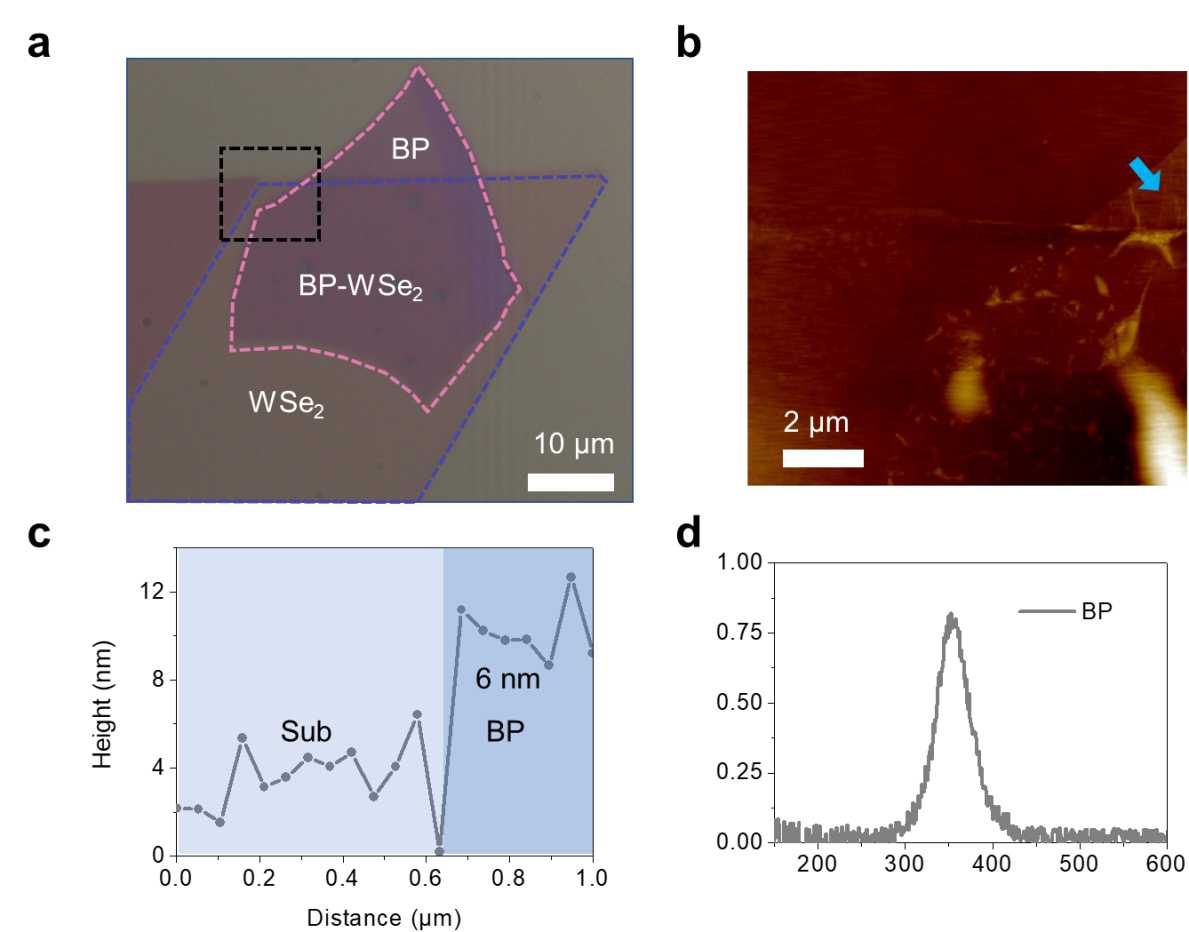


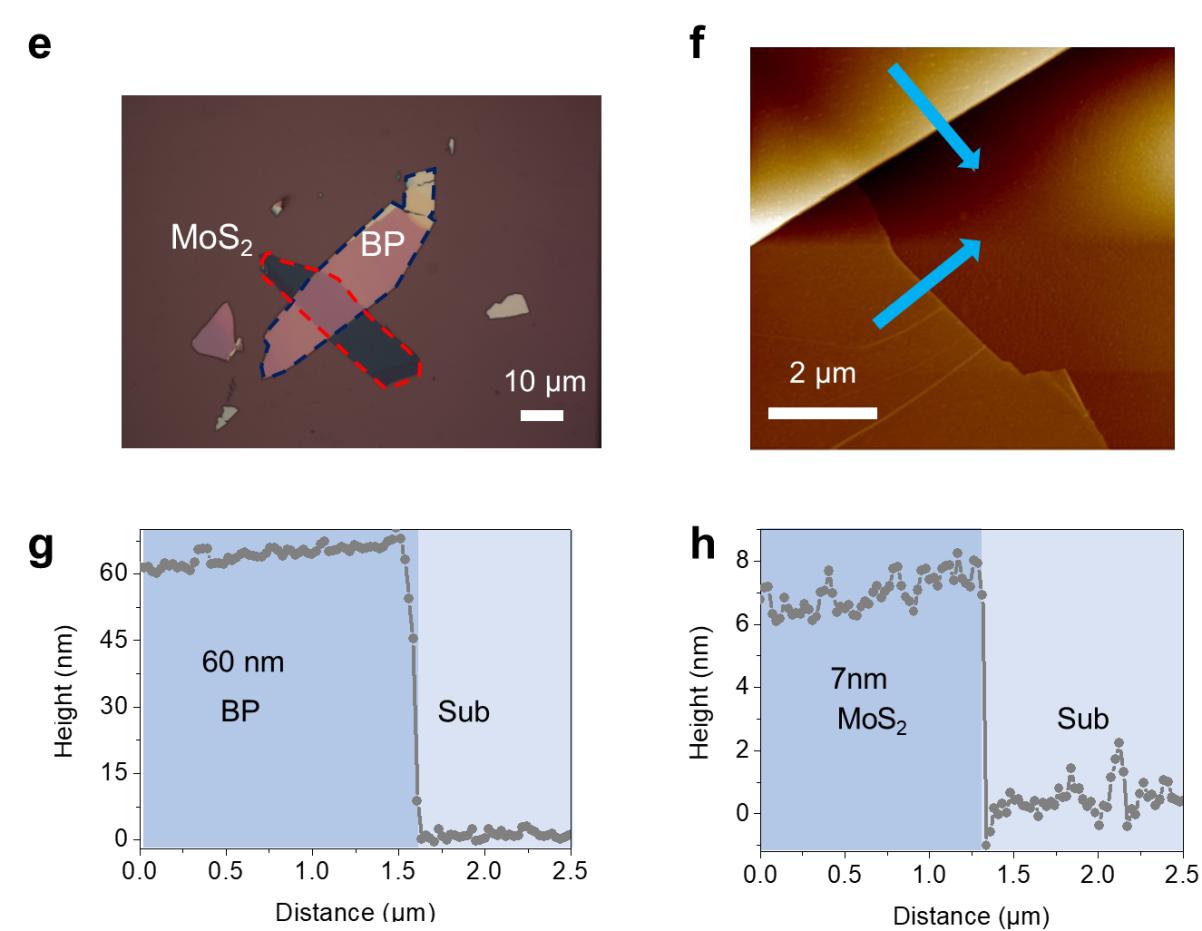


**Figure S2 | Optical images and AFM characterizations**. **a-d**, Optical image (a) and AFM characterizations (b, c) of the BP-WSe_2_ heterostructure shown in the main text. The scale bar is 10 μm. The thickness of BP is about 6 nm, which is consistent with the PL peak position shown in (d)^4^**. e-h**, Optical (e) and AFM (f) images of the BP-MoS_2_ heterostructure. The scale bar in (a) is 10 μm. The thicknesses of BP (g) and MoS_2_ (h) are determined to be 60 and 7 nm, respectively.


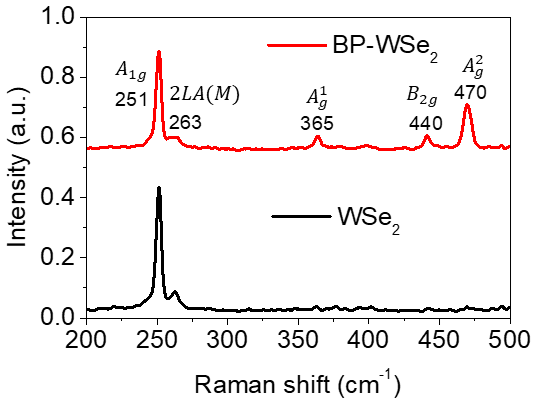


**Figure S3 | Raman spectra of BP-WSe_2_ heterostructure and monolayer WSe_2_**. The 251 cm^-1^ and 263 cm^-1^ peaks correspond to the Raman characteristic peak of monolayer WSe_2_^5^. 365 cm^-1^, 440 cm^-1^, and 470 cm^-1^ correspond to the Raman characteristic peak of BP^6^.


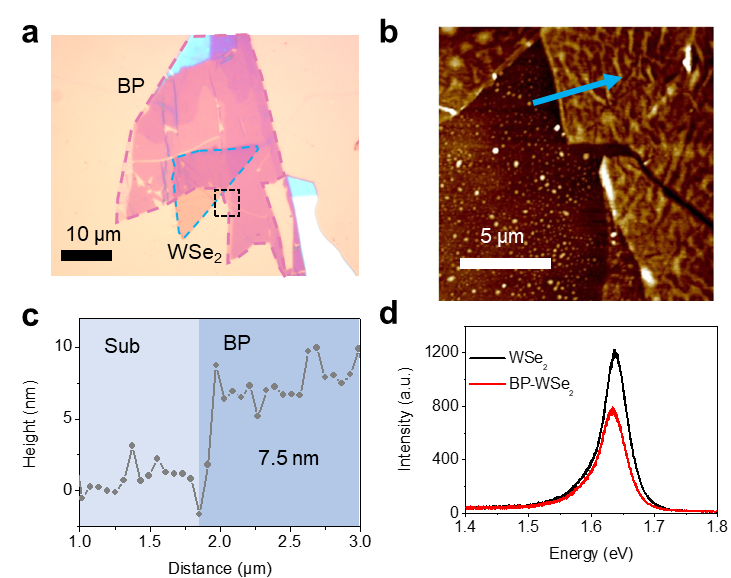


**Figure S4 | Optical characterization of BP-WSe_2_ fabricated in ambient environment. a**, Optical micrograph of BP-WSe_2_. BP and WSe_2_ are outlined with pink and blue dotted lines, respectively. The scale bar is 10 μm. **b**, AFM image of the area enclosed by the black box in (a). **c**, The thickness of BP is determined to be 7.5 nm by AFM measurements. **d**, Visible PL spectra of BP-WSe_2_ heterostructure (red line) and monolayer WSe_2_ alone (black line), respectively.


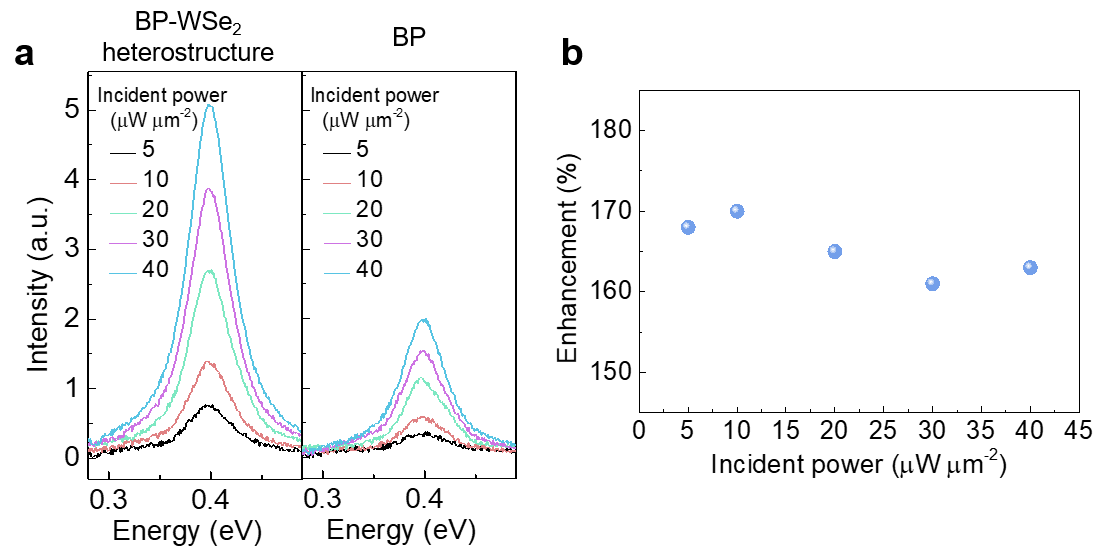


**Figure S5 | Incident-power-dependent enhancement of MIR PL intensity.** (a) The mid-infrared photoluminescence of thin-film BP and BP-WSe_2_ heterostructure at different incident laser powers. (b) The enhancement of mid-infrared photoluminescence at different incident laser powers.


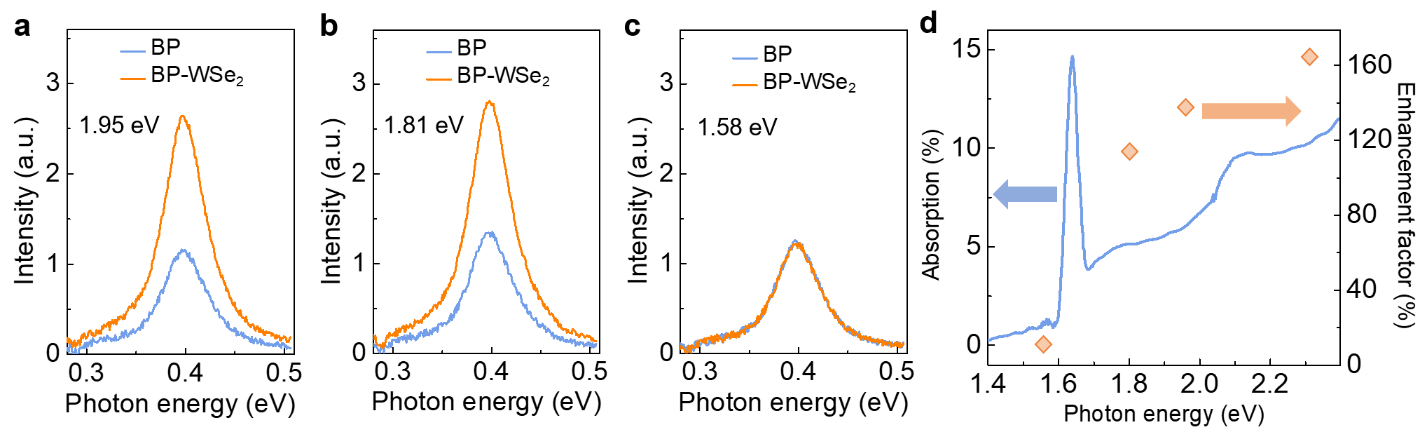


**Figure S6 | Excitation-energy-dependent enhancement factor.** The MIR PL of the 5 nm-thick thin-film BP and BP-WSe_2_ heterostructure under 1.95 eV (a), 1.81 eV (b) and 1.58 eV (c) laser excitation energies. (d) Optical absorption of monolayer WSe_2_ and enhancement factor at different laser excitation energies.


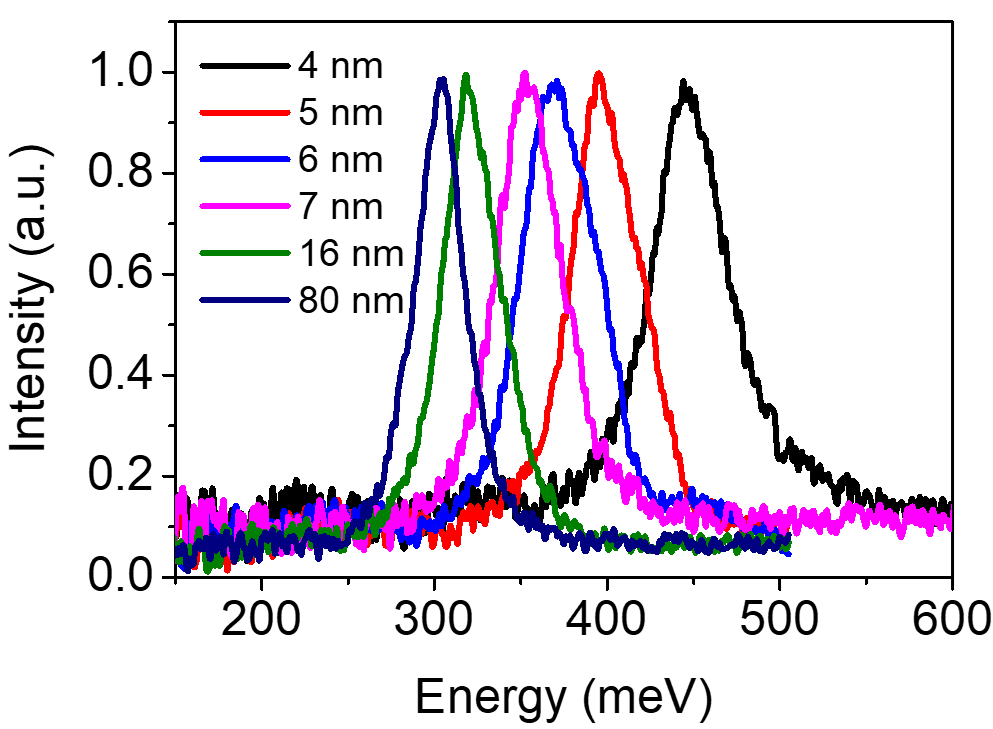


**Figure S7 | Thickness-dependent PL spectra of BP-WSe_2_ heterostructures**. Normalized MIR PL spectra of BP-WSe_2_ heterostructure. The thickness of BP varies from 4 nm to 80 nm and WSe_2_ is monolayer.


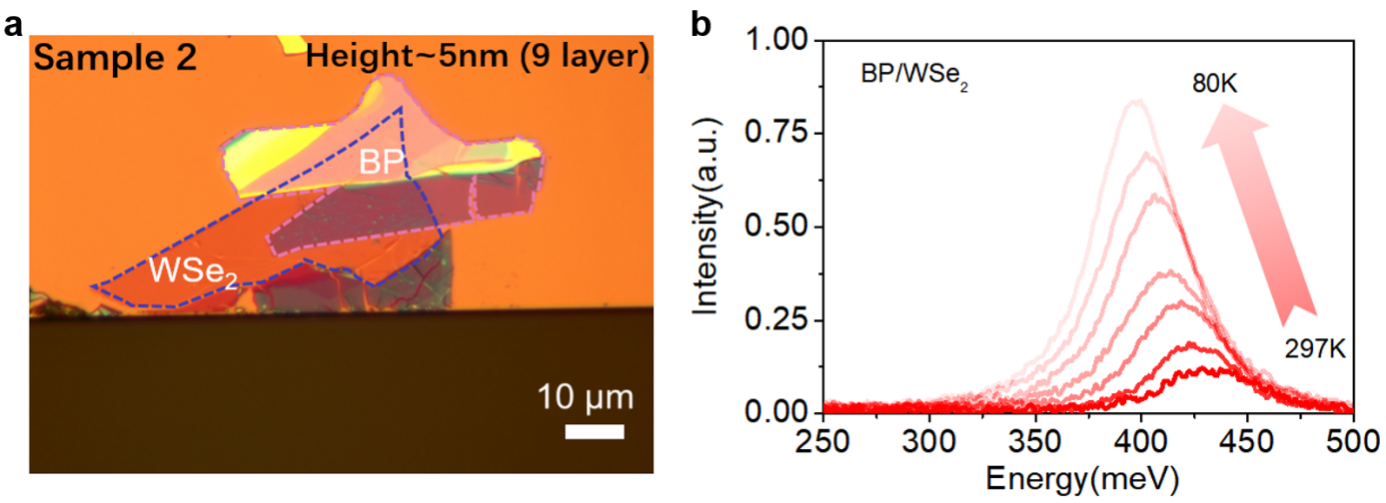


**Figure S8 | Temperature-dependent MIR PL of the 5nm-thick sample.** Optical image (a) and temperature-dependent PL (b) of the 5 nm-thick BP-WSe_2_ heterostructure.


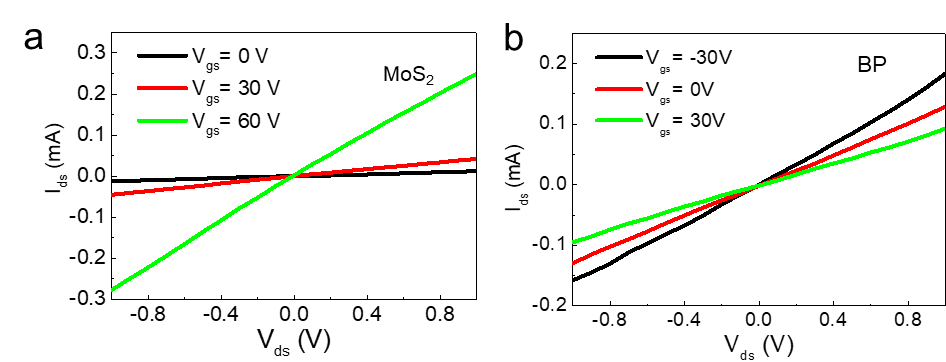


**Figure S9 | Drain -source current-voltage curves for thin-film MoS_2_ and BP**. **a**, **b**, Drain -source current-voltage curves for thin-film MoS_2_ (a) and BP (b). Linear behavior indicates that Ohmic contacts are achieved between metal electrodes and 2D flakes.


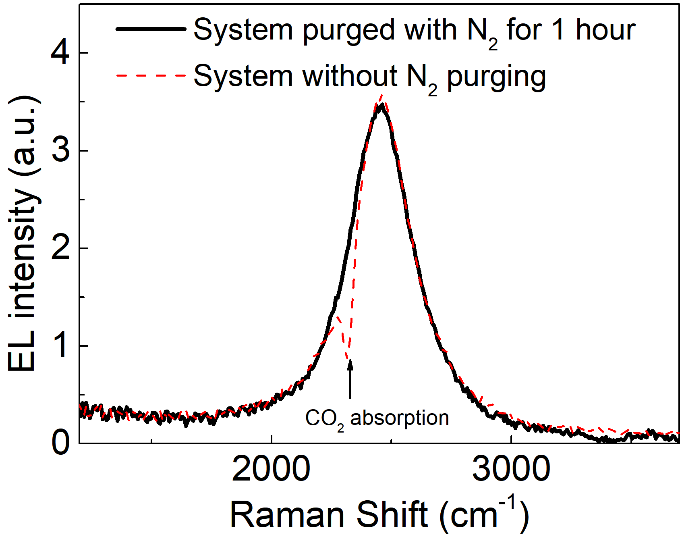


**Figure S10 | Electroluminescence intensity of BP-MoS_2_ heterostructure diode with and without N_2_ purging**.

**Supplementary References**

1. Liao, C., Zhao, Y., Ouyang, G. Strain-modulated band engineering in two-dimensional black phosphorus/MoS_2_ van der Waals heterojunction. *ACS Omega.* **3**, 14641-14649 (2018).

2. Ren, K., Sun, M., Luo, Y., Wang, S., Yu, J., Tang, W. First-principle study of electronic and optical properties of two-dimensional materials-based heterostructures based on transition metal dichalcogenides and boron phosphide. *Appl. Surf. Sci.* **476**, 70-75 (2019).

3. Wang, Q. H., Kalantar-Zadeh, K., Kis, A., Coleman, J. N., Strano, M. S. Electronics and optoelectronics of two-dimensional transition metal dichalcogenides. *Nat. Nanotechnol.* **7**, 699-712 (2012).

4. Chen, C.*,* *et* *al.* Bright mid-infrared photoluminescence from thin-film black phosphorus. *Nano Lett.* **19**, 1488-1493 (2019).

5. Tonndorf, P.*,* *et* *al.* Photoluminescence emission and Raman response of monolayer MoS_2_, MoSe_2_, and WSe_2_. *OSA.* **21**, 4908-4916 (2013).

6. Castellanos-Gomez, A.*,* *et* *al.* Isolation and characterization of few-layer black phosphorus. *2D Mater.* **1**, 2053-1583 (2014).
